# Sinusite ethmoïdo-maxillaire chronique chez un diabétique: penser à l'actinomycose

**DOI:** 10.11604/pamj.2015.20.450.6854

**Published:** 2015-04-30

**Authors:** Madiha Mahfoudhi, Khaled Khamassi

**Affiliations:** 1 Service de Médecine Interne A, Hôpital Charles Nicolle, Tunis, Tunisie; 2 Service ORL, Hôpital Charles Nicolle, Tunis, Tunisie

**Keywords:** Sinusite, diabète, actinomycose, Sinusitis, diabetes, actinomycosis

## Image en medicine

Une sinusite ethmoïdo-maxillaire chronique chez un diabétique est le plus souvent d'origine infectieuse. L'actinomycose doit être évoquée devant une forme unilatérale et trainante. Ce syndrome de sinusite chronique s'accompagne d'une tuméfaction génienne à caractère infiltrant progressif avec fistulisations multiples. Un pus contenant des grains jaunes est évocateur. La culture en milieu anaérobie confirme ce diagnostic. Patiente âgée de 73 ans, diabétique de type 2 depuis 7 ans, a consulté pour épistaxis unilatérale gauche, rhinorrhée purulente ayant un aspect de granulations jaunâtres et algies faciales. Le tout évoluait depuis un mois dans un contexte fébrile. Elle avait des caries dentaires et un diabète déséquilibré. L'endoscopie nasale a trouvé une formation charnue au niveau du méat moyen gauche avec une muqueuse nasale nécrosée. L'IRM du massif facial a révélé un comblement ethmoïdo-maxillo-nasal gauche avec lyse osseuse de la cloison inter-sinuso-nasale et des cornets et infiltration des tissus mous de l'orbite. Plusieurs diagnostics ont été évoqués en particulier une tuberculose, une aspergillose et un lymphome. La culture en milieu anaérobie a confirmé le diagnostic d'actinomycose. Le traitement chirurgical a consisté en une biméatotomie associée à une ethmoïdectomie gauche par voie endonasale, une exérèse des lésions nécrotiques et un drainage sinusien des granules. L'examen anatomopathologique a révélé une nécrose étendue de la muqueuse nasale sans aucun signe de malignité. Le traitement médical s'est basé sur la penicilline G par voie intraveineuse, l’équilibration de son diabète et le soin des caries dentaires. L’évolution était favorable avec un recul de 3 ans.

**Figure 1:**
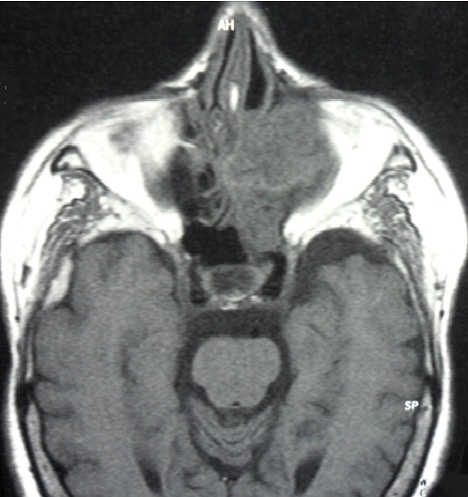
IRM du massif facial (séquence T1): comblement ethmoïdo-maxillaire et nasal gauche en hyposignal T1 avec infiltration de l'orbite

